# 

**Published:** 1904-04

**Authors:** 


### BOOKS RECEIVED.

Diseases of the Intestines. A Textbook for Practitioners and Students of Medicine. By Max Einhorn, M. D., Professor of Medicine at the New York Post-Graduate Medical School and Hospital. Small octavo, pp. 397. Illustrated. Second, revised edition. New York: William Wood & Company. 1904. (Price, $3.00.)

The International Medical Annual. A Yearbook of Treatment and Practitioner's Index. Thirty-two contributors, American and Foreign. Twenty-second year. Octavo, pp. 750. New York and Chicago: E. B. Treat & Co. 1903. (Price, $3.00.)

The Man Who Pleases and the Woman Who Charms. By John A. Cone. Sextodecimo, pp. 131. Hinds & Noble, New York: 1904. (Price, 75 cents.)

A Practical Treatise on Nervous Diseases. For the Medical Student and General Practitioner. By F. Savary Pearce, M. D., Professor of Nervous and Mental Diseases in the Medico-Chirurgical College, Philadelphia.

Octavo, pp. 422. Illustrated. New York and London: D. Appleton & Co. 1904. (Price, $3.00.)

Progressive Medicine, Volume I, March, 1904. A Quarterly Digest of Advances, Discoveries and Improvements in the Medical and Surgical Sciences. Edited by Hobart Amory Hare, M. D., Professor of Therapeutics and Materia Medica in the Jefferson Medical College of Philadelphia. Octavo, 337 pages, 7 illustrations. Lea Brothers & Co. Philadelphia and New York. (Per annum, in four cloth-bound volumes, $9.00; in paper binding, $6.00, carriage paid to any address.)

A Manual of Clinical Diagnosis by means of Microscopical and Chemical Methods. For Students, Hospital Physicians and Practitioners. By Charles E. Simon, M. D., of Baltimore. Fifth edition, thoroughly revised and enlarged. Illustrated with 150 engravings and 22 plates in colors. Octavo, pp. 695. Lea Brothers & Co., Philadelphia and New York. 1904. (Price, $4.00.)

A System of Practical Surgery. By Drs. E. von Bergmann, of Berlin, P. von Bruns, of Tübingen and J. von Mikulicz, of Breslau. Translated and edited by William T. Bull, M. D., Professor of Surgery in the College of Physicians and Surgeons (Columbia University), New York., and Walton Martin, M. D., Instructor in Surgery, College of Physicians and Surgeons, New York. To be complete in five imperial octavo volumes. Volume I, pp. 936. With 361 engravings and 18 plates. Lea Brothers & Co. Philadelphia and New York. 1904. (Price, per volume: Cloth, $6.00; leather, $7.00; half morocco, $8.50.)

Atlas and Epitome of Operative Gynecology. By Dr. O. Schäffer, of Heidelberg. Edited, with additions, by J. Clarence Webster, M. D. (Edin.), Professor of Obstetrics and Gynecology in Rush Medical College, in affiliation with the University of Chicago. With 42 lithographic plates in colors, many text cuts, a number in colors, and 138 pages of text. Philadelphia, New York and London: W. B. Saunders & Company. 1904. (Cloth, $3.00 net.)

Obstetrics for Nurses. By Joseph B. De Lee, M. D., Professor of Obstetrics in the Northwestern University Medical School, Chicago; Lecturer in the Nurses' Training Schools of Mercy, Wesley, Provident, Cook County, and Chicago Lying-in Hospitals. Duodecimo, fully illustrated. Philadelphia, New York and London: W. S. Saunders & Company. 1904. (Cloth, $2.50 net.)

